# Moderate increase in dietary fat induces alterations of microbiota and metabolome along the digestive tract prior to systemic metabolic changes: insights from a pig model

**DOI:** 10.1080/19490976.2025.2587964

**Published:** 2025-12-01

**Authors:** Axel Ranson, Marta Vazquez Gomez, Rohia Alili, Julia Durrafourd, Oriane Vitalis, Paul Taillandier, Clémentine Rebière, Fatiha Merabtene, Eugeni Belda, Daniel Crespo-Piazuelo, Antonio Gonzalez-Bulnes, Geneviève Marcelin, Adil Mardinoglu, Karim Chikh, Tiphaine Le Roy, Karine Clément

**Affiliations:** a Sorbonne Université, Inserm, Nutrition and Obesities: Systemic Approaches, Nutriomics, Paris, France; b CarMeN Laboratory, INSERM, INRAE, Université Claude Bernard Lyon 1, Pierre-Bénite, France; c Lyon GEM Microbiota Study Group, Lyon, France; d IRD, Unité de Modélisation Mathématique et Informatique des Systèmes Complexes, UMMISCO, Sorbonne Université, Bondy, France; e R&D Department, Cuarte S.L., Grupo Jorge, Zaragoza, Spain; f Faculty of Veterinary Sciences, Universidad Cardenal Herrera—CEU, CEU Universities, Alfara del Patriarca, Spain; g Royal Institute of Technology, Stockholm, Sweden; h Assistance-Publique-Hôpitaux de Paris, Nutrition Department, Pitié-Salpêtrière Hospital, Paris, France

**Keywords:** Metabolic diseases, duodenojejunal microbiome, metabolome, small intestine, bile, pig model

## Abstract

The small intestine is a key site for nutrient sensing and host–microbiota interactions, yet how it functionally adapts to dietary changes remains poorly understood. Using a translational porcine model, we investigated the impact of moderate dietary fat increase on the gut microbiota and metabolome across five locations in the digestive tract. Pigs were fed either a low-fat (3%) or a medium-fat (12%) diet for 12 weeks without developing obesity. Multiomics profiling revealed significant dietary effects on bile and duodenojejunal metabolomic profiles, particularly lipid and stachydrine, with notable sex-specific responses. These metabolite shifts were accompanied by segment- and sex-specific changes in microbial communities, including the depletion of metabolically beneficial taxa (e.g., *Limosilactobacillus reuteri* and *Lactobacillus johnsonii*) and the enrichment of bacteria linked to metabolic dysfunction (e.g., *Streptococcus alactolyticus*). In the small intestine lumen, multiple bacterial–metabolite associations correlated with host metabolic markers, suggesting early diet-induced alterations with potential relevance for metabolic disease onset. Our findings position the small intestine as a critical site for early diet-induced microbial and metabolic remodeling, potentially influencing metabolic disease risk and shaping the downstream intestinal environment. This study also underscores the importance of considering both region- and sex-specific responses in diet–microbiota–metabolome research.

## Graphical abstract

**Figure:**
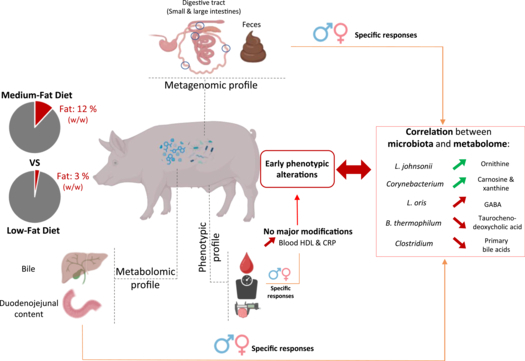

## Introduction

Obesity is a chronic, multifactorial disease that has become a major global health issue, now affecting over one billion individuals worldwide.^1^ Characterized by excessive fat accumulation, obesity significantly increases the risk of numerous comorbidities, including type 2 diabetes, cardiovascular and respiratory diseases, reproductive disorders, certain cancers, and bone fragility.^1^ Understanding its complex etiology and early determinants is critical for developing preventive and therapeutic strategies. The gut microbiota plays a significant role in the development and persistence of obesity and related metabolic diseases.^2^ Changes in microbiota composition can impact host physiology by altering gut barrier function, epithelial integrity, bile acid metabolism, and the expression of genes involved in nutrient absorption and lipid metabolism.^3^^,^^4^ While the gut microbiome is known to be highly sensitive to many environmental factors, including dietary changes, lifestyle factors or drug intake, research has largely focused on fecal samples due to easy accessibility.^5-7^ In this context, the relative abundance of certain microbial taxa, such as *Bifidobacterium spp., Akkermansia muciniphila* and *Lactobacillus rhamnosus*, are frequently associated with beneficial metabolic effects, whereas others, such as *Fusicatenibacter* genus, *Enterobacter cloacae* and *Clostridium ramosum,* may contribute to the onset and persistence of metabolic diseases.^8-13^

However, the fecal microbiota only partially reflects the diversity and function of the microbial communities along the gastrointestinal tract. In particular, the small intestine is a critical but understudied site of host–microbiome interaction.^14^^,^^15^ The small intestine is responsible for the absorption of approximately 90% of dietary energy and plays an essential role in immune, endocrine, and metabolic regulation.^16^ The microbiota at this site is clearly distinct from that of the colon, both in composition and function, and is more dynamic in response to short-term dietary changes.

Among the key players in digestion in the small intestine, bile plays a central role in lipid emulsification and absorption. Indeed, bile acids, synthesized by the liver and secreted into the duodenum, are essential for nutrient absorption, particularly of lipids, and act as key signaling molecules that modulate host metabolism in many organs. After food ingestion, bile acids are released into the small intestine in the form of a complex mixture containing phospholipids and cholesterol. Approximately 95% of bile acids are reabsorbed and recycled to the liver via enterohepatic circulation, while only 5% are excreted.^17^ Bile acids also act as signaling molecules that regulate the host metabolism and inflammation.^18^ Given their interaction with both the intestinal epithelium and the microbiota, bile acids are central to the study of diet-induced metabolic alterations.

Most preclinical studies exploring microbiota and host metabolism interactions have been conducted in rodents. While immensely useful, mouse models differ substantially from humans in gastrointestinal physiology and metabolism. We previously showed that mice, on the contrary to humans, exhibit a relative homogeneity of the intestinal microbiota along the gut, challenging the translational relevance of rodent models for studying the role of the small intestinal microbiota.^19^ This is likely due to the coprophagic behavior of rodents.^20^^,^^21^ In contrast, pigs are marginally coprophagous, with a re-ingestion of feces estimated to maximum 1.5%, versus 35% to 100% in rodents.^22^ In addition, pigs exhibit greater anatomical, physiological, and metabolic similarities to humans and may serve as a relevant model for studying metabolic diseases in link with microbiota changes.^12^^,^^23^^,^^24^ The Iberian pig breed, in particular, presents natural leptin resistance, high appetite, rapid fat accumulation, and a predisposition to features of the metabolic syndrome, making it a valuable model for human obesity and its complications.^25^^,^^26^

Another major challenge in studying the relationship between diet, microbiota, and host metabolic alterations lies in disentangling the specific effects of dietary composition from those driven by the obese state of the host that impairs intestine physiology.^27^ In most animal models, obesity is induced by prolonged high-fat feeding, making it difficult to determine whether the observed alterations in microbiota and metabolic profiles result directly from dietary changes or emerge secondarily due to obesity development. This complexity is amplified by the bidirectional interaction between the host and the microbiota. Not only does diet shape microbial communities, but changes driven by the microbiota can also influence host metabolism and contribute to obesity. Previously, we analyzed the duodenojejunal microbiome and metabolome in individuals with and without obesity and demonstrated that both microbial composition and lipid-derived metabolites from duodenojejunal fluid were significantly associated with dietary intake and clinical obesity-related traits.^19^ These observational findings underscored the difficulty in separating the effects of diet from those of the obese phenotype in human studies and highlighted the importance of investigating the upper gut environment in greater detail.

To disentangle these diet-obesity state effects, we studied the effects of changes in dietary fat intake in the absence of overt obesity. Forty Iberian pigs were fed either a low-fat (3% weight/weight) or a medium-fat (12% weight/weight) diet for 12 weeks—a duration and composition insufficient to induce obesity or major metabolic changes.^28^^,^^29^ Both sexes were represented, as previous work has shown sex-specific responses to high-fat diets in terms of gene expression, fat deposition, and hormonal profiles.^30^^,^^31^ We conducted a comprehensive multiomics analysis including metagenomic sequencing of luminal contents from several intestinal segments and metabolomics of bile and duodenojejunal fluid.

Our findings revealed that dietary fat intake modulation, even in the absence of obesity, induces sex-dependent alterations in bile acid composition and in small intestinal metabolomic profiles. These changes are strongly associated with modifications in the gut microbiota structure, particularly in taxa previously identified as linked to metabolic health.

## Materials and methods

### Maintenance of animals and experimentation

The research was carried out on a commercial farm (Paraje El ladrillar – Cuarte S.L., Alcaraceros, Spain) according to the European Union Directive and the Spanish Policy for Animal Protection RD53/2013 and following the standard handling of pig production. The Committee of Ethics in Animal Research of the Universidad CEU Cardenal Herrera (CEU) assessed and approved the experimental procedures (CEEA 23/01 Report). Animals were managed in accordance with standard practices in pig production, including identification with electronic chips and housing indoors under controlled temperatures.

The purebred Iberian pigs were housed in collective pens with free access to water and fed, *ad libitum*, with a pig grower diet based on wheat, barley, and pork fat (total fat 3% weight/weight), the usual fat concentration in a maintenance diet for Iberian pigs, supplemented with recommended levels of amino acids, vitamins, and minerals (Tables S1 and S2). No antibiotics were used from weaning, at 4 weeks old, to the end of the trial. At 10 weeks of age, pigs were randomized by dividing equally by sex, into two treatment groups fed, *ad libitum*, either a low-fat diet (LFD) or a medium-fat diet (MFD). Pigs with the LFD (*n* = 20) continued with the initial diet with 3% of fat, while pigs with the MFD (*n* = 20) switched to a diet containing higher percentage of pork fat (12% weight/weight), the highest concentration avoiding significant weight gain over the duration of the experiment, significant changes in diet texture, feed rancidity and potential animal refusal, during 12 weeks. During the experiment, some losses occurred, reducing the total number of pigs available for final sampling. First, one LFD-fed female died in the first week. At week 10, two animals (one MFD-fed female and one MFD-fed male) were excluded because they needed an antibiotic treatment. An MFD male developed meningitis, causing its weight to drop drastically, leading to its exclusion from the study.

Body weight was measured at weeks 0, 6, 8, and 10 after randomization and on the day before the sacrifice. Morphometric measurements, such as the thickness of the fat subcutaneous superficial and deep layer, the length of the head and body, and the circumference of the thorax and abdomen, were collected at weeks 0 and 6 after randomization and before the sacrifice. Blood samples were collected from the ophthalmic venous sinus with EDTA-coated tubes after a 14-h fasting period at week 0 and the day prior to the sacrifice. Plasma was obtained by centrifugation of the blood for 15 min at 2500 × g.

All the animals were euthanized with a penetrating captive bolt gun, followed by carotid artery severing. The kidneys, heart, liver, spleen, lungs and carcass were dissected and weighed. Feces, intestinal luminal content, intestine samples, bile, liver, adipose tissue and muscle were collected, and all the samples were snap-frozen in dry ice and subsequently kept at −80 °C until further analysis.

### Metabolomic analyses

Approximately 100 mg of each diet was diluted 1:10 with ethanol-PBS (85/15 v/v), 1.4 mm ceramic beads were added, and the samples were homogenized with a Precellys 24 (Bertin Technologies, Montigny-le-Bretonneux, France) by shaking at 5800 rpm three times for 30 sec, interspersed with a 30 s pause. Then the homogenized samples were centrifuged at 2000 × g for 2 min at 2 °C–4 °C. The supernatants were transferred and centrifuged again at 2000 × g for 2 min. The supernatants were then transferred to a new tube and centrifuged at 10,000 × g for 5 min at 2 °C–4 °C just before using MxP 500 Quant XL kit.

The bile samples were used without prior treatment, while the duodenojejunal contents were centrifuged at 10,000 × g for 5 min at 4 °C, and 2 × 10 µL of the supernatant was used for metabolomic analysis.

Metabolomics analyses were performed at the METANUTRIBIOTA Metabolomics platform (LYON, France). The samples were analyzed in a randomized order, using the commercial MxP Quant 500 XL kit (Biocrates Life Science AG, Innsbruck, Austria, https://biocrates.com/mxp-quant-500-kit/ (accessed on 22 February 2024) on a XEVO TQ-XS® instrument coupled to a UPLC (Waters, Milford, Massachusetts, USA).

This targeted assay allows the detection and quantification of up to 1019 metabolites from 39 biochemical classes, using liquid chromatography (LC) and flow injection analysis (FIA) MS/MS, according to the manufacturer's instructions. The analysis was carried out using a Waters ACQUITY PREMIER LC system coupled to a Waters XEVO-TQXS triple quadrupole mass spectrometer equipped with an electrospray ionization (ESI) source (Waters, Saint-Quentin-en-Yvelines, France). Serum samples, external standards, and quality control samples were loaded onto 96-well filter plates, which were preloaded with internal standards. The workflow consisted of derivatization, extraction, and dilution before injection. Alkaloids, amine oxides, amino acids, amino acid-related metabolites, bile acids, biogenic amines, carboxylic acids, cresols, fatty acids, hormones, indole derivatives, nucleobase-related metabolites, vitamins, and cofactors were detected via MS after liquid chromatography using an MxP Quant 500 column. Acylcarnitines, lysophosphatidic, phosphatidic acids, lysophosphatidylcholines, phosphatidylcholines, lysophosphatidylethanolamines, phosphatidylethanolamines, lysophophatidylglycerols, phosphatidylglycerols, lysophosphatidylinositols, phosphatidylinositols, lysophosphatidylserines, phosphatidylserines, sphinganines and sphingosines, sphinganine and sphingosine phosphates, sphingomyelins, ceramides, dihydroceramides, hexosylceramides, dihexosylceramides, trihexosylceramides, cholesteryl esters, monoglycerides, diglycerides, and triglycerides were processed via FIA-MS/MS method. Data were collected with MassLynx® software (Waters, Milford, Massachusetts, USA) and analyzed with Biocrates WebIDQ™ software (Biocrates Life Sciences AG, Innsbruck, Austria). The limit of detection of each metabolite was based on the Quant 500 XL kit methodology in accordance with the manufacturer's instructions.

### Metagenomic analysis

DNA was extracted from 150 ± 30 mg of samples for feces and ascending colon luminal content and from 400 ± 40 mg of ileal, jejunal and duodenal luminal content. DNA extraction was performed using NucleoMag DNA Microbiome Kit (Macherey-Nagel, Vertrieb GmbH & Co. Kg). Two cycles of chemical- and mechanical-lysis were performed with a Precellys 24 (Bertin Technologies, Montigny-le-Bretonneux, France). Then, the protocol was performed according to the manufacturer's instructions, except that 250 instead of 500 µL of sample lysate was used for automated robot for DNA purification using paramagnetic beads (Auto-Pure96, Nucleic Acid Purification System Hangzhou Allsheng Instruments Co., Ltd. Hangzhou, Zhejiang, China). The purity ratio and DNA quantity were controlled (NanoDrop and Qubit, Thermo Fisher).

DNA libraries were prepared from 150 ng of DNA, using the Illumina DNA PCR-Free Library Prep Kit according to the manufacturer's instructions. The sequencing of the samples was performed on a NovaSeq 6000 sequencer by Szaomics Company (Istanbul, Turkey). The raw sequencing data (.bcl) were demultiplexed and converted to FASTQ with DRAGEN Software, v4.2.7, and the data were delivered in fastq format using Illumina 1.8 quality scores.

To account for variations in sequencing depth across samples within segments, random sampling of a defined number of read pairs using seqtk was used. Within each segment, this number was defined by the number of pairs of reads of the sample with the lowest number, after eliminating samples with an insufficient sequencing depth, that is, the samples with an end of slope above 0.075 marker gene per read pair on the rarefaction curves (Figure S2h–j).

Metagenomic analyses were performed using the bioBakery tools.^32^ Read-level quality control was performed using KneadData with default settings, including quality filtering with Trimmomatic (SLIDINGWINDOW:4:20 minLEN:75) and removal of porcine contaminant sequences with bowtie2 in very sensitive mode vs. assembly reference.

Taxonomic profiling was performed using MetaPhlAn4-catalog vs mpa_vJan21_CHOCOPhlAnSGB_202103 reference database (21,978 species-level genome bins (SGB) derived from a reference gene catalog of 5.1 million taxonomic markers).^33^

### Bacteria quantification in intestinal luminal content and feces by quantitative PCR

The DNA samples were used for amplification of the V3-V4 region of the 16S rRNA gene. Quantitative PCR was performed by mixing 0.2 µL of each forward (5ʹ-ACTCCTACGGGAGGCAGCAG-3ʹ) and reverse (5ʹ-ATTACCGCGGCTGCTGG-3ʹ) primer at a concentration of 10 µM, with 2.05 µL of water, 5 µL of Sybr green mix (Applied, 4385612), and 0.05 µL of BSA 50 mg/mL (Thermo Fisher, AM2616). 7.5 µL of this mixture was added to 2.5 µL of DNA, diluted to 0.1 ng/µL in a 384-well PCR plate (MicroAmp, 4483285). The PCR cycling conditions were an initial denaturing step of 95 °C for 20 s, followed by 40 cycles of 95 °C for 3 s and 60 °C for 30 s, followed by a step of 95 °C for 15 s, 60 °C for 60 s and a final step of 95 °C for 15 s. A standard curve was included on each plate by diluting genomic DNA from *Turicimonas muris* pure culture of known bacterial concentrations. The bacterial concentration in the intestinal samples and feces was then calculated from the quantified amount obtained via qPCR and taking into account the various dilution factors along the DNA purification process and the amount of sample that was weighed prior to DNA extraction.

### Plasma glucose and lipids homeostasis

Glucose and urea, as well as parameters related to the lipid profile, total cholesterol, high-density lipoprotein (HDL) cholesterol, low-density lipoprotein (LDL) cholesterol, nonesterified fatty acids (NEFAs), and triglycerides, were measured in the plasma at CEU Cardenal Herrera University (Valencia, Spain). Assays were performed with a clinical chemistry analyzer (Konelab 20i Chemistry Analyzer, Thermo Fisher Scientific, Madrid, Spain) using the corresponding kit for each enzymatic method (SPINREACT, Sant Esteve de Bas, Spain; except for the NEFA kit, Fujifilm Wako, PALEX MEDICAL, S.A.U., Sant Cugat del Valles, Spain).

### Plasma insulin quantification

The assay was carried out according to the kit HUman/swine/canine insulin ELISA kit (RK00302, ABclonal Technology, Germany) instructions, using 40 µL of plasma instead of 100 µL. Measurements were performed in technical and biological duplicates. This measurement was used to calculate HOMA-IR using the formula: HOMA-IR = $\frac{\mathrm{Insulin}\;({µU\;\mathrm{mL}}^{-1})\times \mathrm{blood}\;\mathrm{glucose}\;({\mathrm{mg}\;\mathrm{dL}}^{-1})}{405}$​.

### Plasma CRP quantification

The assay was carried out according to the kit Sandwich ELISA Kit for C-Reactive Protein (ABIN67390937, ANTIBODIES ONLINE, USA) instructions with 100 µL of plasma. Measurements were performed in duplicates**.**

### Lipocalin II quantification in duodenojejunal content and feces

The assay was carried out according to the kit Sandwich ELISA Kit for Lipocalin 2 (ABIN6957506, ANTIBODIES ONLINE, USA) instructions. The feces and duodenojejunal contents were diluted 1:10 in PBS−/− and centrifuged at 12,000 × g at 4 °C for 15 min, and the supernatant was used for the assays. Measurements were performed in duplicates**.**

### Liver histology

The livers were fixed in 4% paraformaldehyde in PBS for 24 h and then embedded in paraffin blocks. Sections were then cut at 5 µm with a microtome and stained with hematoxylin and eosin (H&E) before viewing under a light microscope. The staining procedure involved immersing dry slides in xylene twice for 10 min each, followed by 100% ethanol for 5 min. The slides were then immersed in a series of decreasing ethanol concentrations: 100%, 75%, and 30% and finally PBS, each for 5 min. Staining was performed by immersing slides in hematoxylin for 3 min, followed by a 30-s water rinse, eosin for 30 s, and another 30 s water rinse. After staining, the slides were quickly dehydrated in 100% ethanol and xylene and then mounted using Eukitt mounting medium. Imaging was performed on an Echo Revolve microscope.

### Statistical and bioinformatic analysis

Statistical analyses were performed with R (version 4.3.3) (https://www.R-project.org/). The statistical tests used are indicated in the legend to each figure.

All sequencing data preprocessing and analysis steps were performed using R (version 4.3.3) or Python (version 3.7.12). The *Vegan* v2.6.4 R package was used for ecological analyses of the metagenomic profiles. Alpha diversity was determined from a random sampling of defined read pair numbers, defined for each segment, per sample with seqtk to account for variations in sequencing depth across samples. The COBRApy (Constraint-Based Reconstruction and Analysis for Python) v0.26.2 package was used to study bacterial metabolism with AGORA2, using VMH database (https://www.vmh.life/#microbes/).^34^ The Ggtree v3.14.0 R package was used to generate cladograms. The Venn diagram v1.7.3 R package was used to generate Venn diagrams. The corrplot v0.95 R package was used to generate correlation matrices. The ggplot2 v3.5.1 R package was used to generate the graphics, particularly boxplots. The Tidyr, v1.3.1, Dplyr v1.1.4, Perm v1.0.0.4, Effsize v0.8.1, Coin v1.4.3, Stringr v1.5.1, Writexl v1.5.1, Ggrepel v0.9.6, Grid v4.4.2, RColorBrewer v1.1.3, Tidyverse v2.0.0, and Ggpubr v0.6.0 packages were used to analyze, visualize and for the aesthetics of the data and figures. The complete code is available on request.

## Results

### Impact of diet on physiological and metabolic parameters in pigs

A medium-fat diet did not induce significant increases in total body weight and length, body fat depot amount or distribution, glucose and insulin levels and HOMA-IR, lipocalin 2 in feces and duodenojejunal content, or total plasma cholesterol (Table 1). Furthermore, the liver lipid content, assessed by histological examination, showed no evidence of steatosis in MFD-fed animals (data not shown). Organ weights were also similar, with the exception of liver weight, which was lower in MFD-fed females (Table S3). Analysis of fasting plasma lipid levels revealed only a moderate increase in circulating HDL-cholesterol levels in pigs fed an MFD in both males and females and a slight increase in C-reactive protein (CRP) in females fed the MFD (*p* ≤ 0.05; Table 1) without reaching levels indicative of significant inflammation.^35^ These findings confirm that the dietary intervention, while modifying lipid intake, did not induce overt metabolic alterations or obesity-related phenotypes. The metabolic neutrality of the increase in fat content during this timeframe provided a basis for investigating the impact of dietary fat content on the composition of the gut microbiota and intestinal metabolite profiles, independently of the effects of obesity or of the development of metabolic disease.

**Table 1.** Morphometric and metabolic characteristics​.

	Females			Males		
	LFD (n = 9)	MFD (*n* = 9)	*p*-value	LFD (n = 10)	MFD (*n* = 8)	*p*-value
**Morphometric measures**						
Body weight (kg)	62.01 (±7.02)	62.58 (±10.07)	0.931	69.79 (±7.57)	69.11 (±7.02)	0.965
Body Length (cm)	95.56 (±3.94)	94.22 (±4.58)	0.655	97.23 (±3.77)	97.38 (±5.01)	0.822
Head length (cm)	26.11 (±1.08)	26.33 (±1.00)	0.820	27.30 (±0.67)	28.00 (±1.85)	0.321
Fat subcutaneous superficial layer (cm)	9.87 (±1.01)	10.57 (±2.71)	0.267	10.16 (±3.87)	12.33 (±2.11)	0.284
Fat subcutaneous deep layer (cm)	13.11 (±3.51)	14.73 (±4.09)	0.507	13.93 (±6.13)	14.48 (±3.96)	0.722
Abdominal thickness (cm)	4.08 (±0.55)	4.11 (±1.32)	0.859	4.48 (±1.82)	5.19 (±2.30)	0.464
Muscle thickness (cm)	3.01 (±0.49)	3.34 (±0.76)	0.420	3.68 (±0.54)	3.11 (±1.31)	0.556
Abdominal circumference (cm)	103.11 (±7.39)	104.61 (±.,81)	0.859	106.23 (±7.21)	106.13 (±7.16)	0.964
Torso circumference (cm)	95.22 (±4.6)	98.94 (±5.57)	0.114	104.30 (±6.25)	102.06 (±6.06)	0.422
**Plasma glucose and lipid homeostasis**						
Fasting glucose (mg/dL)	104.67 (±13.47)	116.51 (±21.51)	0.190	108.41 (±8.04)	113.57 (±12.45)	0.360
HOMA-IR index	1.96 (±0.72)	2.90 (±1.19)	0.050	2.28 (±0.83)	2.20 (±0.61)	0.460
Fasting triglycerides (mg/dL)	50.29 (±14.37)	56.20 (±23.57)	0.931	63.80 (±14.52)	61.13 (±17.94)	0.696
Fasting NEFA (mmol/L)	0.21 (±0.10)	0.14 (±0.08)	0.222	0.23 (±0.13)	0.17 (±0.11)	0.173
Fasting total cholesterol (mg/dL)	104.17 (±6.78)	109.56 (±15.33)	0.546	116.57 (±16.11)	116.85 (±15.82)	0.965
Fasting HDL-cholesterol (mg/dL)	41.11 (±3.46)	53.36 (±9.58)	0.002	58.68 (±5.93)	66.91 (±8.46)	0.027
Fasting LDL-cholesterol (mg/dL)	52.18 (±13.18)	46.45 (±6.42)	0.387	55.84 (±12.54)	46.91 (±10.98)	0.101
Fasting urea (mg/dL)	31.54 (±4.81)	29.80 (±3.64)	0.605	34.22 (±9.01)	30.09 (±11.41)	0.122
**Inflammation markers**						
CRP (µg/mL) – plasma	10.66 (±3.19)	15.67 (±5.29)	0.019	6.12 (±4.74)	4.34(±2.53)	0.633
Lipocalin 2 (ng/g) – duodenojejunal content	43.26 (±49.32)	20.22 (±23.84)	0.408	41.36 (±71.70)	53.94 (±82.19)	0.585
Lipocalin 2 (ng/g) – feces	46.44 (±51.18)	36.53 (±30.56)	0.694	49.70 (±44.04)	41.38 (±32.95)	0.689

Abbreviations: CRP: C-reactive protein; HDL: high-density lipoproteins; HOMA-IR: homeostatic model assessment for insulin resistance; LDL: low-density lipoproteins; LFD: low-fat diet; MFD: medium-fat diet; NEFA: non-esterified fatty acids. Note: Values correspond to the mean of each pig group with the standard error of the mean (SEM). *p*-values correspond to a Wilcoxon test between LFD and MFD groups of same-sex pigs. The *p*-values in bold indicate a statistically significant difference between the two diets for the same sex.

### Effect of diet on biliary and duodenojejunal metabolome profiles

**Figure 1.**
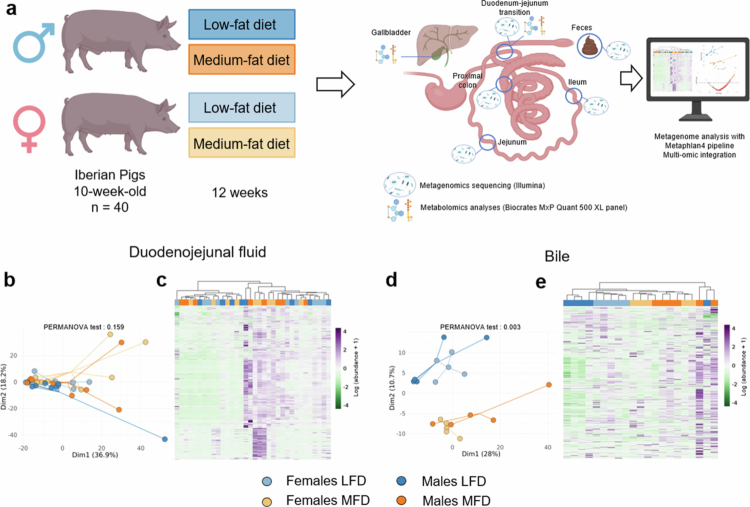
Experimental design and metabolomic profiling of pig duodenojejunal fluid and bile. (a) Experimental design. Forty Iberian pigs (n = 10 per group) were fed either a low-fat diet (LFD, 3%) or a medium-fat diet (MFD, 12%) for 12 weeks, starting at 10 weeks old, with equal representation of males and females. (b and d) Principal component analysis (PCA) plots showing the metabolite profiles of duodenojejunal fluid (b) and bile (d) for individual pigs across the four experimental groups. Each dot represents a single pig, and lines connect each pig to the centroid of its group. A PERMANOVA test is performed on the Euclidean distance, and the exact test result is indicated above each figure. (c and e) Heatmaps of the relative abundance of metabolites detected in duodenojejunal fluid (c) and bile (e). The columns represent individual pigs; the rows represent measured metabolites. Hierarchical clustering was performed based on metabolomic profiles, and dendrograms reflect similarities between pigs and conditions for metabolites. Color codes: light blue = female LFD, blue = male LFD, light orange = female MFD, orange = male MFD.

In order to better understand the interplay between the diet, the proximal small intestine physiology, the proximal small intestine microbiome and the bile that is secreted into the duodenal lumen, we first performed a multitargeted metabolomic analysis of the low-fat and the medium-fat diets as well as the bile and duodenojejunal (DJ) contents of the pigs (Figure 1a). Principal component analysis (PCA) of DJ fluid metabolome revealed no clear clustering based on either diet or sex, suggesting relatively modest variation in the luminal metabolome at the proximal intestine site (Figure 1b,c). In contrast, bile metabolite profiles clearly showed distinct clustering, validated by a PERMANOVA test (*p* = 0.003), with a stronger effect of diet than sex (Figure 1d,e). Nonetheless, sex also influenced the bile metabolome, and differences in the metabolic response to dietary fat were evident between males and females in univariate analyses (Table S4). Together, these findings suggest that while the DJ fluid metabolome remains moderately affected in response to moderate changes in dietary fat, bile composition appeared more sensitive to both dietary and sex-dependent metabolomic changes.

### Diet-induced shifts in metabolite classes and sex-specific responses

**Figure 2.**
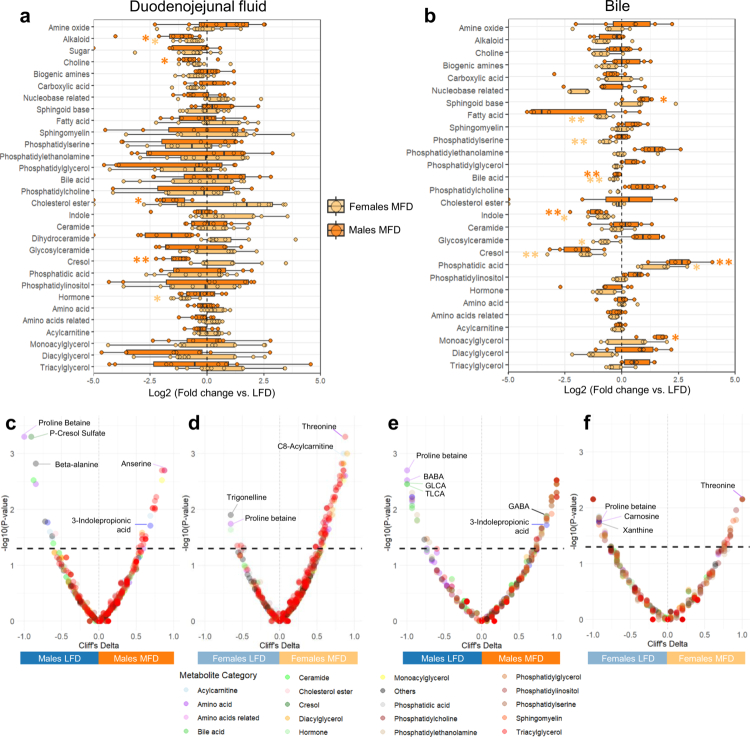
Diet- and sex-dependent differences in duodenojejunal and biliary metabolites. (a and b) Log2 fold changes in metabolite abundance (MFD vs. LFD groups) grouped by chemical class in duodenojejunal fluid (a) and bile (b), shown separately for males and females (boxplots). Columns represent the sum of all metabolites for each category, and p-values from Wilcoxon rank-sum tests comparing diet groups within each sex are shown on boxplots (**p ≤ 0.01, *p ≤ 0.05). The absence of asterisks indicates non-significance. (c–f) Volcano plots of Cliff's delta effect size analyses comparing LFD and MFD pigs: duodenojejunal fluid for males (c) and females (d) and bile for males (e) and females (f). Each dot represents a metabolite, with the color indicating its chemical class. The x-axis represents Cliff's delta values (positive values = enrichment in the MFD group, negative values = enrichment in the LFD group), and the y-axis represents –log10 (*p*-values). *p*-values are calculated from a permutation test (*n* = 2,000) with Cliff's delta as the effect measure. The horizontal dotted line marks the significance threshold (*p* = 0.05). Color code: light blue = female LFD, blue = male LFD, light orange = female MFD, orange = male MFD. Abbreviations: GABA, gamma-aminobutyric acid; BABA, beta-aminobutyric acid; GLCA, glycolithocholic acid; TLCA, taurolithocholic acid.

To further dissect the impact of diet, we examined changes in specific metabolite classes in both DJ fluid and bile, taking into account the false discovery rate (FDR). Several classes were significantly altered by diet, with a more pronounced effect observed in the bile (Figure 2a,b). Interestingly, some changes were consistent across compartments; for example, a reduction in cresol levels in both DJ fluid and bile in males fed the MFD; while most were compartment-specific (Figure S1). Notably, alkaloids decreased only in DJ fluid of MFD pigs, while phosphatidic acids increased. Indole derivatives and bile acids decreased exclusively in the bile of MFD pigs (Figure 2a,b). Importantly, diet also elicited sex-specific responses. In bile, only males exhibited an increase in monoacylglycerols and sphingoid bases, whereas only females showed a decrease in phosphatidylserine concentrations (Figure 2b).

While the metabolites affected differ between sexes, the overall trends reflected a consistent diet-induced modulation of lipid-related metabolites, except for a few categories, such as dihydroceramide in DJ fluid or glycosylceramide in bile (Figure 2a,b). To identify individual metabolites driving these patterns, we conducted Cliff delta analyses. Several metabolites were significantly associated with diet type, including a strong enrichment in numerous triacylglycerols in DJ fluid of MFD pigs despite no change in the total amount of triglycerides (Figure 2c,d). This trend was also observed in bile, particularly in males, although fewer triacylglycerol species were detected in this compartment (Figure 2e,f). Beyond lipid classes, other specific metabolites were differentially abundant depending on diet and sex. Notably, proline-betaine and trigonelline levels were elevated in the DJ fluid of both male and female pigs fed with a LFD (*p* ≤ 0.05; Figure 2d). In males, the LFD was associated with higher levels of stachydrine (proline-betaine), an indole sulfate in both DJ fluid and bile, of *p*-cresol sulfate, ornithine and choline in DJ fluid. Looking at bile, secondary bile acids (glycolithocholic acid (GLCA), taurolithocholic acid (TLCA), glycodeoxycholic acid (GDCA), and glycoursodeoxycholic acid (GUDCA)) were increased in the LFD group. MFD was linked to 3-indolepropionic acid in both DJ fluid and bile, anserine in DJ fluid and gamma-aminobutyric acid in bile (*p* ≤ 0.05; Figure 2c,e; Table S5a,c). In females, differences were also observed in DJ fluid and bile, with the appearance of nitrotyrosine in MFD females (Figure S1d). The MFD group was associated with lower levels of proline-betaine and higher levels of threonine in both DJ fluid and bile. It showed also elevated levels of hydroxyproline and creatinine in DJ fluid and decreased in p-cresol sulfate, carnosine, xanthine and primary (glycochenodeoxycholic acid (GCDCA), taurochenodeoxycholic acid (TCDCA)) and secondary (GLCA) bile acids in bile (*p* ≤ 0.05; Figure 2d,f; Table S5d).

Finally, sex differences were particularly observed in DJ fluid. Volcano plot profiles showed that, in females, a large fraction of metabolites were significantly enriched in MFD group (72 vs. 6 in LFD groups), whereas in males, associations were more evenly distributed between the LFD and MFD groups (Figure 2c,d, Table S5a,b). For bile, there was an inverse dynamic in the number of metabolites enriched in both sexes fed the MFD diet (94 metabolites in males vs. 28 in females) (Table S5c,d). These findings highlight both diet- and sex-specific signatures in the intestinal and biliary metabolomes.

### Diet and sex influence microbial community structure along the digestive tract

**Figure 3.**
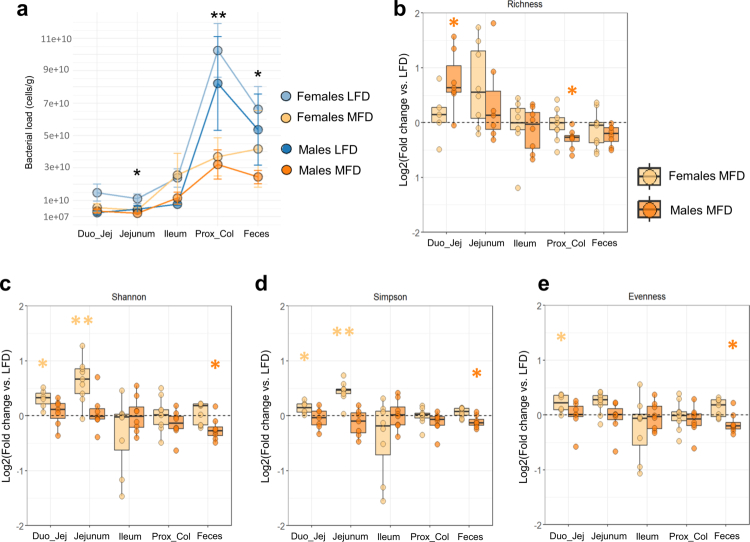
Bacterial load and alpha diversity along the digestive tract in pigs. (a) Total bacterial load across five segments of the digestive tract (duodenojejunal content, jejunal content, ileal content, proximal colon content, and feces) in each experimental group. The dots represent the group mean ± SEM. Asterisks indicate statistically significant differences, by Wilcoxon tests, comparing LFD and MFD groups are indicated above each plot between LFD and MFD females (**p ≤ 0.01, *p ≤ 0.05). (b–e) Alpha diversity indices comparing the MFD and LFD groups in each gut segment stratified by sex: richness (b), Shannon index (c), Simpson index (d), and evenness (e). Boxplots represent the log2-fold change of MFD groups relative to LFD groups within each sex. The samples were normalized based on their rarefaction curves (Figure S2b–g). Wilcoxon rank-sum *p*-values comparing LFD and MFD groups are indicated above each plot (**p ≤ 0.01, *p ≤ 0.05). The absence of asterisks indicates non-significance. Color code: light blue = female low-fat diet (LFD), blue = male LFD, light orange = female medium-fat diet (MFD), and orange = male MFD. Abbreviations: Duo_Jej, duodenojejunal content; Prox_col, Proximal colon content.

Bacterial load and metagenomic profiles of DJ, jejunal (middle), ileum (terminal), proximal colon contents and feces were analyzed in order to determine the effects of changes in dietary lipids on the pig microbiome all along the digestive tract. The bacterial load, assessed via quantitative PCR targeting a conserved region of the 16S rRNA gene, increased progressively along the digestive tract across all experimental groups (Figure 3a). While no significant differences in bacterial load were observed between LFD and MFD males, MFD females exhibited a significant reduction in bacterial load in the jejunal content (*p* ≤ 0.05), proximal colon content (*p* ≤ 0.01), and feces (*p* ≤ 0.05) compared to their LFD counterparts (Figure 3a).

As expected, the sequencing depth of host-decontaminated reads was highest in the distal segments of the digestive tract, which contain fewer host-derived sequences (Figure S2). This was highlighted by statistically different slopes at the end of the rarefaction curves between the different gut segments (Figure S2), indicating uneven taxonomic coverage. Consequently, samples with an end of slope on rarefaction curves above 0.075 were excluded from the analysis, and α-diversity analyses were conducted on a “segment-by-segment” basis rather than across the entire dataset to avoid biases arising from differential taxonomic coverage (Figure S2h). Alpha diversity analyses revealed diet-induced changes across all five gut segments in both sexes (Figure 3b–e; Table S6). Specifically, MFD-fed animals had a higher alpha diversity in the proximal small intestine content (duodenojejunum, jejunum), similar alpha-diversity in the ileum and decreased alpha-diversity in the proximal colon and feces in comparison with LFD-fed animals. This change in alpha diversity was associated with the disappearance of *Escherichia coli*, *Veillonella caviae* (only in males), *Blautia wexlerae* and several *Staphylococcus* (only in females) species and the appearance of *Pseudoscardovia suis*, *Dialister succinatiphilus*, *Sharpea porci*, *Bifidobacterium pseudolongum* and *Aeriscardovia aeriphila* species in the proximal small intestine of the MFD groups (Table S6a–c). In the distal segments (proximal colon and feces), the MFD groups were depleted in several *Roseburia*, such *as Roseburia faecis*, *Corynebacterium*, and *Negativibacillus massiliensis*, and we noted the appearance of *Pseudoscardovia* and *D. succinatiphilus* (as observed in proximal segments) but also of several *Clostridium* species (Table S6d,e). Additionally, a greater effect size on alpha-diversity indices is noted in the small intestine for females and in the proximal colon and feces for males, again demonstrating the different responses to diet between sexes (Figure 3b–e).

**Figure 4.**
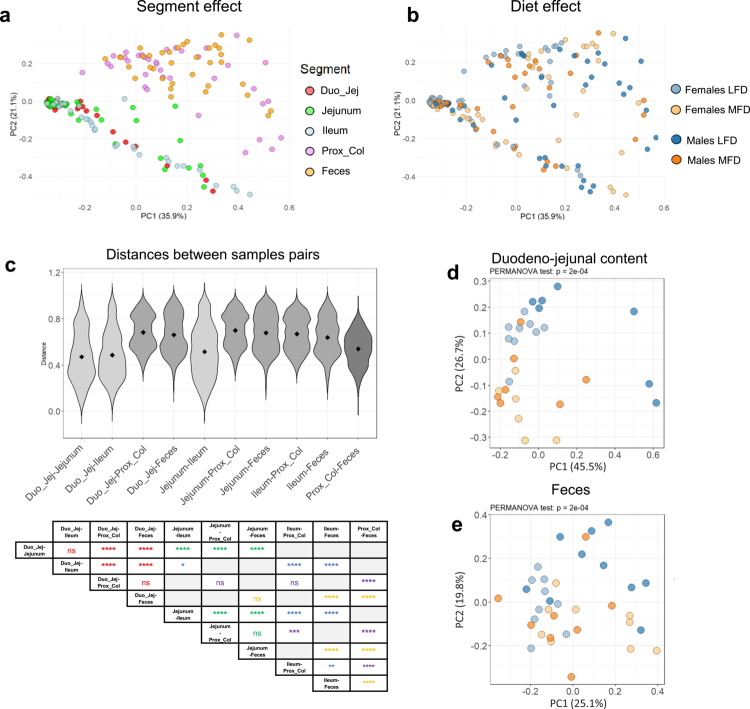
Diet and segment effect on the microbial diversity of the 5 segments of the digestive tract. (a and b) Principal coordinate analysis (PCoA), which is based on Bray‒Curtis dissimilarity metrics, shows the distance in the bacterial community of the 5 segments of the pigs of each group, colored by segment (a) or by group (b). (c) The violin plots show the distance between the points of two segments in light gray if the segments are part of the small intestine, in dark gray if one segment is part of the small intestine and the other of the distal segments, and in black if both segments are distal. The table shows the statistical differences between the different comparisons, the color code corresponds to the segment compared, and the asterisk corresponds to the result of a Tukey test adjusted after an ANOVA test (****p ≤ 0.0001, ***p ≤ 0.001, **p ≤ 0.01, *p ≤ 0.05, ns = non-significant). The statistical results are shown only for studying comparison pairs with a common segment. (d and e) PCoA, based on Bray‒Curtis dissimilarity metrics, showing the difference between the four groups for the duodenojejunum (d) and feces(e). The result of a PERMANOVA test on the 4 groups is shown at the top of the graph. Color codes: group: light blue = female LFD, blue = male LFD, light orange = female MFD, orange = male MFD. Segment: red = duodenojejunum (Duo_Jej), green = jejunum, light blue = ileum, purple = proximal colon (Prox_Col), orange = feces.

Beta diversity analysis based on the Bray-Curtis distance revealed that the anatomical segment of the gut exerts a stronger influence on microbial composition than the diet. Indeed, microbial profiles clustered primarily according to whether they originated from proximal (small intestine) or distal (colon/feces) segments across both the LFD and MFD groups (Figure 4a,b). The higher similarity of microbial profiles from different segments of the small intestine and between the proximal colon and feces was confirmed by the analysis of distances between sample pairs from different segments (Figure 4c). Nevertheless, distinct clustering by sex and diet was observed within each segment, except in the ileum, where no clear group separation occurred (Figures 4d,e; S3a–c). Assessment of intragroup distances between sample pairs showed minimal differences in microbiome dispersion between LFD and MFD males, except in the ileum (Figure S3d). In contrast, MFD females exhibited significantly increased intragroup distances across all segments except the jejunum (Figure S3e). However, intragroup dispersion was higher in distal segments than in proximal parts of the gut (Figure S3d). When comparing inter-group distances (LFD vs. MFD groups) within each segment, the largest differences were observed in the distal gut, particularly in the proximal colon and feces, with the exception of the jejunum in males (Figure S3f,g).

These findings indicate that even a moderate change in dietary fat content leads to substantial restructuring of the microbial communities along the gut, albeit with segment- and sex-specific patterns. We next sought to characterize the specific microbial taxa driving these changes.

### Taxonomic shifts along the digestive tract in response to diet

**Figure 5.**
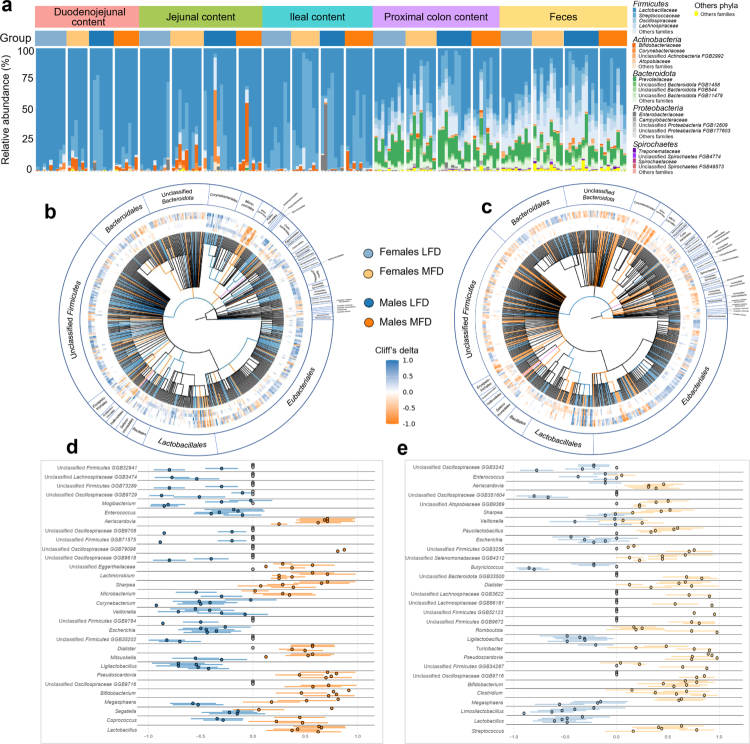
Diet-induced taxonomic changes in the gut microbiota along the digestive tract. (a) Stacked bar plots showing the microbial composition across gut segments and experimental groups. The plots represent the relative abundance of the four most represented families within the five most abundant phyla: Firmicutes (blue), Actinobacteria (orange), Bacteroidota (green), Proteobacteria (gray) and Spirochaetota (purple). The remaining families and phyla are grouped under “Other families” and “Other phyla” (yellow). Segment and group identities are indicated above each histogram. (b and c) Cladograms depicting the 500 most diet-responsive bacterial taxa based on Cliff's delta effect size, shown separately for males (b) and females (c). Each node represents a taxonomic level from kingdom to species (kingdom, phylum, class, order, family, genus, and species). Colors indicate the direction of statistically significant, from a permutation test (*n* = 2,000) with Cliff's delta as an effect measure, of change in MFD pigs across the five segments: orange = increased in the MFD group; blue = decreased in the MFD group; violet = both increased and decreased across different segments. Heatmaps in (b and c): Diet-associated changes in the abundance of the same 500 bacterial taxa across gut segments (from inside to outside: duodenojejunum, jejunum, ileum, proximal colon, and feces). The orange squares indicate enrichment in the MFD groups; the blue squares indicate enrichment in the LFD groups. Taxa are ordered by their taxonomic classification along the outer circle. (d and e) Forest plots of the 30 most diet-modified bacterial genera for males (d) and females (e), ranked by Cliff's delta effect size. Each point represents the Cliff's delta of a genus in a specific segment (duodenojejunum, jejunum, ileum, proximal colon, feces). Orange dots = enrichment in the MFD group; blue dots = enrichment in the LFD group; gray dots = not detected in that segment. The horizontal bars indicate variability across segments (standard deviation), obtained using the bootstrap method, at 95%. Color codes: group: light blue = female LFD, blue = male LFD, light orange = female MFD, orange = male MFD. Segment: red = duodenojejunum (Duo_Jej), green = jejunum, light blue = ileum, purple = proximal colon (Prox_col), orange = feces.

Across all the gut segments, the microbial communities were predominantly composed of Firmicutes, especially members of the *Lactobacillaceae* family, with *Lactobacillus amylovorus* being the most abundant species, although strictly anaerobic families such as *Oscillospiraceae* and *Lachnospiraceae* families had a significantly lower relative abundance in the small intestine (Figures 5a; S4). In contrast, Bacteroidota were largely restricted to distal segments, the proximal colon and feces (Figure 5a).

Dietary intervention induced a compositional shift in the microbiota that extended to the kingdom level (Figure 5b,c). Notably, in MFD females, a decrease in Bacteria and a corresponding increase in Archaea were observed (Figure 5c). At the phylum and class levels, Actinobacteria were enriched in both MFD males and females, rising from 1% to 8% in jejunum, which was driven primarily by an increase in Bifidobacteriales relative abundance, while Bacilli and Firmicutes were more abundant in pigs fed LFD (Figure 5b,c). At the genus level, *Enterococcus, Veillonella, and Corynebacterium* were decreased by MFD in the duodenojejunal content of males. *Ligilactobacillus* genus was decreased in the duodenojejunal content of MFD-fed animals, where it decreased from 1.39% to 0.23% in females and from 0.63% to 0.25% in males, while *Dialister, Aeriscardovia, Pseudoscardovia* and *Bifidobacterium* genera were enriched in the MFD groups (Figure 5d,e).

At finer taxonomic resolution, the relative abundance of *Lactobacillus* species was significantly and positively associated with LFD in female pigs. This finding aligns with the observed reduction in *L. amylovorus* and *L. johnsonii* relative abundance, which decreased from 6.83% to 0.43% in the jejunum, in MFD females in comparison to LFD females (Figure 5e; Figure S4). Interestingly, while *Limosilactobacillus reuteri, Lactobacillus delbrueckii* and *L. johnsonii* decreased in both sexes under MFD, and *Oscillibacter valericigenes, P. radai* and *P. suis* increased in both MFD groups, *L. amylovorus* increased consistently across all segments in MFD males, highlighting again a sex-specific response of the microbiome to the diet (Figure S4). Low-abundance taxa also exhibited segment- and sex-specific shifts. For example, Proteobacteria were selectively altered only in females, and Eggerthellales in males (Figure 5b–e). Moreover, Corynebacteriales abundance decreased significantly in MFD males, while remaining were stable in females. A noteworthy finding is that microbial changes across intestinal segments were opposite for specific taxa (Figure 5b–e). For example, *Megasphaera*, rising from 0.02% to 0.49% in the jejunum and falling from 1.24% to 0.42% in the feces of MFD males, and *Segatella* (only for males) are enriched in proximal segments of MFD pigs and depleted in distal ones, reflecting a differential effect of the diet depending on the digestive tract location.

These results reinforce the notion that diet influences the gut microbial composition throughout the digestive tract, with divergent responses depending on both anatomical location and sex.

### Microbiota–metabolite interactions reflect early diet-induced functional shifts

**Figure 6.**
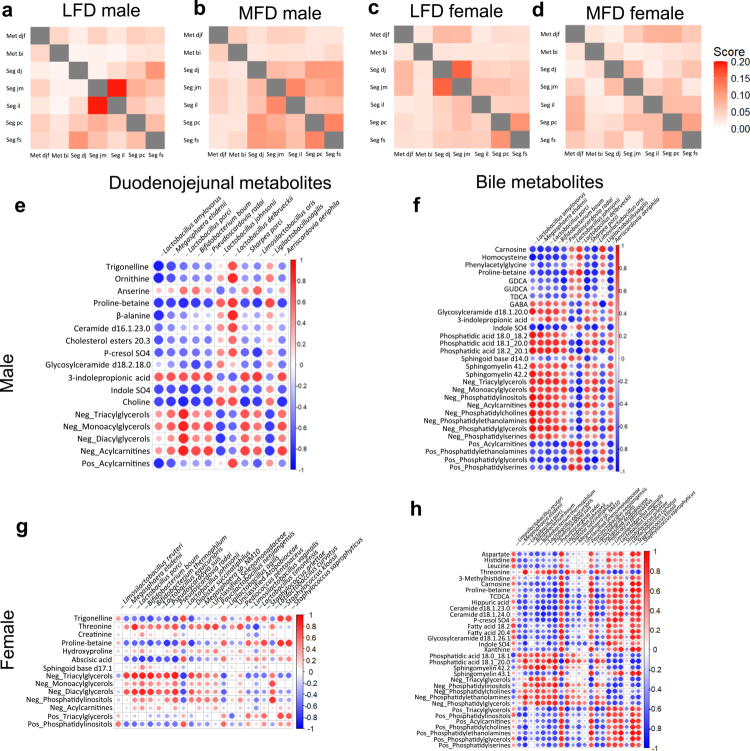
Diet alters microbiota–metabolite correlation networks in a sex-specific manner. (a–d) Summary of statistically significant correlations between metagenomic and metabolomic datasets across five gut segments in the LFD and MFD groups for males (a and b) and females (c and d). Each matrix represents the proportion of significant correlations (*p* ≤ 0.05 of the Spearman correlation test) relative to the total number of possible pairwise associations between the microbiota and metabolites per condition. (e–h) Correlation heatmaps showing interactions between bacteria significantly altered by diet in DJ for males (e and g) and females (f and h) and metabolites significantly altered by diet in DJ (e and f) and bile (g and h). Only metabolites known to be linked to bacterial metabolism (VMH and MiMeDB databases) were included. Dots represent the results of a Spearman correlation test: red = positive, blue = negative. The dot size indicates the strength of the correlation. When many metabolites of the same metabolite category were present, they were grouped according to the Cliff's delta value (Cliff's delta > 0 = Pos, Cliff's delta < 0 = Neg). Abbreviations: Met djf, metabolome duodenojejunal fluid; Met bi, metabolome bile; Seg dj, microbiota of the duodenojejunal segment; Seg jm, microbiota of the jejunal segment; Seg il, microbiota of the ileum segment; Seg pc, microbiota of the proximal colon segment; Seg fs, fecal microbiota; GDCA, glycodeoxycholic acid; GUDCA, glycorsodeoxycholic acid; TDCA, taurodeoxycholic acid; GABA, γ-aminobutyric acid; TCDCA, taurochenodeoxycholic acid.

Given the observed changes in both microbiota and metabolite profiles depending on diet and animal sex, we next assessed whether these alterations were functionally linked. We correlated the concentration of each metabolite with the relative abundance of each microbial taxon and computed the proportion of statistically significant correlations to evaluate the strength of the associations between the metabolome and microbiome in each localization of the digestive tract (Figure 6a–d).

Again, we observed that diet modulates the network of associations along the digestive tract, with sex-specific patterns (Figure 6a–d). In males, MFD feeding resulted in an overall increase in significant correlations between the microbiota and metabolites in different intestinal segments, as well as between microorganisms in different segments except for the ileum with the jejunum, suggesting a possible cascade of diet-induced microbial changes throughout the digestive tract (Figure 6a,b). In females, correlation patterns within each segment of the gut shifted markedly depending on diet. Under LFD conditions, strong associations were found between duodenojejunal metabolites and the microbiota in proximal segments (duodenojejunum and jejunum), whereas in MFD-fed animals, these associations were instead observed in more distal segments (ileum and proximal colon) (Figure 6c,d).

To explore specific microbial–metabolite interactions, we focused on bacteria and metabolites that were significantly altered by diet in the DJ fluid and bile, in males and in females (Figure 6e–h). In order to detect a biologically explicable effect, only metabolites with known links to bacterial metabolism, based on the Virtual Metabolic Human (VMH) and MiMeDB databases, were included. We observed stronger and more consistent correlations between bile metabolites and microbiota composition than between DJ fluid metabolites and microbiota composition, which is coherent with our previous observations. Several associations observed were consistent with the microbial metabolic functions predicted by the use of AGORA2 on bacteria significantly modified by the diet, such as positive correlations between *L. delbrueckii* or *L. johnsonii* relative abundance and ornithine concentration, or between *Lactobacillus oris* and the production of gamma-aminobutyric acid (GABA), as well as negative correlations between *Bifidobacterium thermophilum* and taurochenodeoxycholic acid, which are linked to its transformation by this bacterial species (Figure 6e–h). These results highlight functional shifts that may underlie diet-driven alterations in host metabolism, although limited by the scope of existing annotations of bacterial genomes and experimentally demonstrated metabolic activities of gut microbes.

Despite the absence of overt diet-induced physiological changes in these pigs, we identified significant correlations between microbiota composition, metabolite concentrations, and host metabolic parameters in MFD-fed pigs (Table 1; Table S7). For example, we found that a reduction in *Coprococcus catus* abundance in the duodenojejunum was associated with increased plasma insulin, which itself correlated with lower levels of indole derivatives (e.g., indole-3-acetic acid, indoxyl sulfate) and xanthine and higher levels of bile acids.

Similarly, several *Corynebacterium* species, previously associated with the LFD groups, were negatively correlated with the marker of insulin resistance index, HOMA-IR, and subcutaneous adipose tissue thickness (both deep and superficial layers), which in turn showed strong associations with ceramides, choline, and secondary bile acids such as glycolithocholic acid and glycoursodeoxycholic acid (Table S7). Furthermore, in females*, L. delbrueckii* is inversely correlated with the level of lipocalin II in the DJ fluid content and with several phosphatidic acids, ceramides and fatty acids in the DJ fluid while *Pseudoscardovia suis* is positively associated with lipocalin II level linked to the concentrations of phosphatidylserines, phosphatidylinositols and triacylglycerols in the DJ fluid. Thousands of significant correlations (*p* ≤ 0.05) were found for each sex between the physiological data, despite their slight modifications, the metagenomic data of the small intestine and the metabolomic data, suggesting that a crucial link of the small intestinal microbiome in modifying phenotypes correlated with metabolic diseases (Table S7).

These findings suggest that even in the absence of obesity, a moderately high-fat diet induces early, coordinated shifts in the small intestinal metabolome and microbiota composition that can be linked to metabolic traits related to body composition and glucose homeostasis.

## Discussion

Our study demonstrates that even a moderate increase in dietary fat, without inducing obesity and major metabolic deterioration, can coordinately reshape the gut microbiota and metabolome along the digestive tract, with sex- and segment-specific signatures. Using a translational Iberian pig model, we deciphered early ecosystem-level changes in the small intestine that may prime the host for future metabolic disturbances.

To our knowledge, this is the first study assessing multisegment metagenomic and metabolomic responses to moderate dietary fat changes in this translational porcine model.^26^ Only another study in male Iberian pigs, preventing sex gap analysis, had assessed the evolution of microbiota composition between several intestinal segments, but sequencing was limited at the gender level, limiting analysis.^36^ Previous studies, including those from our group performed in humans, have described significant associations between diet characteristics, the small intestinal microbiota and host obesity and metabolic status.^10^^,^^19^^,^^37^ One of the major challenges in microbiome‒diet studies in chronic disease is separating the effects of lifestyle (including diet) from those of the disease state *per se*, thus necessitating the use of animal models to investigate these cause-and-effect relationships. Moreover, many human studies are cross-sectional and confounded by pre-existing metabolic alterations or other factors, such as medications, in subjects with advanced chronic diseases.^38^ On the other hand, rodents' coprophagic behavior may limit their utility in modeling the upper gut microbiota in response to dietary modifications.^20^^,^^21^

The choice of the two diets used in the present study was based on both the total fat content and the distribution of fatty acids, which play distinct roles in host metabolic health. A LFD (3% weight/weight fat) has previously been used in pigs without deleterious metabolic effects, and human studies have reported comparable outcomes under LFD.^39^^,^^40^ In contrast, the 12% w/w fat diet is characterized by a higher amount of both saturated fatty acids (SFA) and monounsaturated fatty acids (MUFA) (respectively 15.5 and 19.1% of energy), placing it in the range of SFA intake of a typical occidental diet (10%−16% of daily energy intake), rich in SFA of animal origin, and within the range of MUFA intake of a Mediterranean-type diet (15%−20% of energy intake), which is enriched in MUFAs from olive oil.^41–45^ While the elevated SFA content is associated with the development of metabolic disorders, the high MUFA fraction may partly explain the increase in HDL observed in the MFD groups (Table 1).^41^^,^^46^ Thus, these two diets were selected to model, in pigs, a comparison between a diet considered metabolically favorable and another more deleterious in humans, without inducing overt obesity and significant metabolic deterioration, thereby allowing the discrimination of dietary effects from obesity-related confounders.

Here, using Iberian pigs, a model with strong anatomical and metabolic similarities to humans and low coprophagic behavior, we were able to isolate the effects of a moderately increased fat intake prior to overt metabolic dysfunction.^26^ Indeed, although pigs fed a MFD for 12 weeks did not develop marked obesity, insulin resistance or inflammation, we observed significant changes in bile composition, duodenojejunal metabolites, microbial diversity and bacterial communities. Interestingly, several of these features have been shown to be associated with overt metabolic disorders or bio-clinical traits characterizing these disorders.

Metabolomic profiling revealed that bile is a compartment that is more sensitive than the proximal small intestine luminal content to dietary fat modulation, with striking compositional changes and some sex-specific differences. Notably, we found in MFD-fed pigs an increase in phosphatidic acids (PAs), alongside a significant reduction in conjugated secondary bile acids such as glycolithocholic acid and taurolithocholic acid, which are molecules with known anti-inflammatory and metabolic regulatory properties via FXR and TGR5 signaling.^47^ This reduction in secondary bile acids has already been observed in obese pigs, although without reaching statistical significance.^48^ However, the quantification of all bile acid species in pigs remains challenging due to the diversity of conjugated and minor bile acids, which may influence the total measured concentration as well as the ratio between primary and secondary bile acids. The detection of secondary bile acids in the bile is explained by their enterohepatic recirculation^49^^,^^50^: after their microbial conversion from primary bile acids in the intestine, up to 95% of luminal secondary bile acids are reabsorbed and reconjugated in the liver before being resecreted into the bile.^17^^,^^51^ Therefore, changes in their biliary concentrations may reflect either alterations in their intestinal microbial transformation or dysregulation of the intestinal absorption and hepatic recirculation processes. PAs are not only intermediates in lipid metabolism and membrane biosynthesis but also act as bioactive lipids involved in intracellular signaling pathways regulating mTOR activation, cell growth, and inflammation.^52^^,^^53^ Elevated levels of PAs in bile may reflect an adaptive response to increased lipid intake, possibly linked to enhanced lipoprotein assembly or altered bile secretion dynamics. However, excessive or sustained increases in PAs could perturb bile fluidity or signaling, with downstream effects on intestinal lipid handling and barrier function. While bile is a critical interface for dietary lipid digestion and microbial interaction, its compositional remodeling in response to early dietary changes has been largely overlooked in metabolic research.^54^

These bile alterations in MFD-fed animals were paralleled by shifts in duodenojejunal metabolites (e.g., elevated triacylglycerols, 3-indolepropionic acid, and nitrotyrosine) and suggest early disruption in lipid handling, redox balance, and microbial activity occurring prior to measurable systemic changes.^55^^,^^56^ Among the metabolites altered by increased dietary fat, compounds with known protective functions were depleted in both the bile and duodenojejunal fluid of MFD-fed pigs. This was the case for glycolithocholic acid, which was also significantly reduced in DJ fluid.^57^ Similarly, p-cresol sulfate levels were lower in both compartments. While it is often considered a uremic toxin associated with metabolic disease, studies have focused on its urinary excretion. Interestingly, p-cresol itself modulates small intestinal transit and gut hormone secretion,^58^ and paradoxically, p-cresol sulfate has been shown to reduce body fat mass in animal models,^59^ also suggesting context-dependent effects. The observed elevation of 3-indolepropionic acid, a microbiota-derived antioxidant and epithelial barrier stabilizer, may represent a compensatory microbial response.^56^ Other compounds were depleted in MFD pigs, such as stachydrine, also called proline-betaine, which is a naturally occurring alkaloid with reported anti-inflammatory and antifibrotic properties via modulation of TGF-β signaling,^60^ or trigonelline, which is a niacin-related alkaloid abundant in legumes and coffee and is associated with improved insulin sensitivity and reduced adiposity,^61^ as well as bile acid derivatives that play a crucial role in protecting against metabolic and inflammatory diseases.^54^^,^^62^^,^^63^ These metabolites have been inversely associated with metabolic disease, reinforcing the notion that moderate dietary fat increases can perturb a metabolite landscape previously linked to metabolic health. Some metabolites are uniquely modified in one of the compartments, such as trigonelline in duodenojejunal fluid and xanthine in bile, implying an effect of microbiota and/or intestinal cells on the abundance of these metabolites. Furthermore, several microbial metabolites modified by the diet were described as crucial in controlling intestinal barrier efficacy by acting on the epithelial permeability or mucus production.^56^^,^^64^^,^^65^ For example, a decrease in ornithine and an increase, although not statistically significant, in spermidine and putrescine in both bile and duodenojejunal fluid of MFD groups, implying an increase in polyamine synthesis from ornithine^66^ or a decrease in indole derivatives and bile acids in MFD groups.

These metabolomic changes were accompanied by a depletion of key commensal taxa throughout the digestive tract in MFD-fed pigs, which are known to have beneficial metabolic roles, including *L. reuteri,*^67^ *L. johnsonii*,^68^^,^^69^*B. wexlerae,*^70^ *Corynebacterium,*^71^ and *L. amylovorus,* particularly in females.^72^ Based on oral administration in rodents, these species were described to support mucosal health and host metabolic regulation. Their depletion may signal early disruption of intestinal homeostasis. We also observed, in MFD-fed pigs, the appearance or enrichment of bacteria considered associated with metabolic alterations, such as *Streptococcus alactolyticus,*^73^^,^^74^ *B. pseudolongum*^74^ or *Oscillibacter valericigenes.*^75^ In mice and rats, these species have been shown to promote inflammatory process and to be associated with increased blood levels of triglycerides, LDL-cholesterol and CRP. Although *Bifidobacterium* is typically associated with metabolic health benefits, linked to an increased fiber intake, and used as a probiotic, the increase observed in our study is not entirely unexpected.^76^ First, the *Bifidobacterium* genus is highly diverse, and not all *Bifidobacterium* species are able to metabolize all dietary fibers, as this capacity largely depends on the degree of polymerization of the substrates.^77^ For example*, in vivo* studies in rats have shown that fibers with a low degree of polymerization, such as fructans or fructo-oligosaccharides, do not systematically alter the abundance of this genus in the gut.^77^ In addition, the MFD used in our study is rich in SFA, with similarities to a WD in humans. Several studies in both mice and humans have reported that SFA consumption, particularly diets enriched with coconut oil, can increase the abundance of *Bifidobacterium* linked to indole derivatives decrease, which is consistent with our results.^78–81^ Finally, clinical studies have shown that patients with obesity or NAFLD present a higher abundance of this genus in the gut microbiota, and experimental work has demonstrated that supplementation with specific species, such as *B. breve* or *B. longum* in the context of a WD can improve the phenotype.^19^^,^^81^ Taken together, these findings are consistent with the notion that diets rich in SFA may promote shifts in *Bifidobacterium* abundance. Whether such changes are beneficial or detrimental likely depends on the specific species and substrates involved and cannot be directly inferred from our data.

Rare bacteria are also differentially modified according to the gut segment, such as *M. elsdenii*, increased in the small intestine and decreased in distal segments by MFD in males and females. This bacterium is essentially studied for its role in preventing acidosis and for performing lactate fermentation in ruminants.^82^ Its decrease in the large intestine can be explained by lower starch intake in MFD groups, but for the small intestine, the lack of literature limits our interpretation. However, a study conducted in humans has observed *Megasphaera* increase in the small intestine of people with obesity, supporting our results, although the number of patients and statistical analysis is limited.^83^ It is noteworthy that despite the decrease of indole derivatives and bacteria capable of synthetizing it in MFD pig groups, such as *Lactobacillus* or *E. coli,*^84^ there is an increase of some bacteria that can produce specific indole derivatives, such as species of *Clostridium* genus, which are able to produce 3-indolepropionic acid.^56^^,^^85^ It suggests a precise reorganization of metabolites and not just a complete modification of one category of metabolites.

Such reshaping of the gut ecosystem by the MFD occurred with increased microbial alpha-diversity in the small intestine but decreased diversity in the colon and feces, patterns that mirror those observed in individuals with obesity and metabolic diseases.^10^^,^^19^^,^^86^ While higher diversity is often interpreted as a hallmark of a healthy microbiome, emerging evidence suggests that in the small intestine, increased richness may reflect a loss of niche specificity and impaired microbial regulation, features increasingly associated with dysbiosis. Conversely, reduced diversity in the distal gut has been consistently linked to metabolic impairment and a shift toward pro-inflammatory microbial communities.^87^

Our results highlight marked sex-specific responses to dietary fat. MFD induced greater microbiota and metabolite shifts in the small intestine of females, whereas changes were more pronounced in the colon and feces of male pigs. These patterns are consistent with known sex differences in lipid metabolism and fat distribution, which are influenced in part by hormonal factors such as DHEA.^31^^,^^88-90^ Notably, certain taxa were specifically altered in females, including a reduction in *L. amylovorus* and enrichment of *Streptococcus alactolyticus*, a species previously linked to obesity and metabolic disturbances in rodents.^73^^,^^74^ A modest increase in CRP, a marker of inflammation associated with type II diabetes and cardiovascular disease in particular, was observed only in females, aligning with clinical data showing higher CRP levels in women across health and disease states.^91^ Although this increase remained within the physiological ranges observed in healthy pigs (around 15 µg/mL), its sex specificity further supports the translational relevance of the Iberian pig model for studying early metabolic alterations.^35^^,^^92^

Diet also reshaped microbiota‒metabolite relationships, as revealed by correlation analyses across gut segments. In MFD males, microbial–metabolite correlations became more structured across compartments, suggesting coordinated ecosystem responses. In contrast, females exhibited a redistribution of associations, shifting from proximal to distal segments. This difference could be explained by the difference in transit speed between the two sexes.^93^^,^^94^ Several correlations were supported by predicted microbial metabolic functions (VMH/AGORA2), including *L. johnsonii* and *L. delbrueckii* with ornithine, *L. oris* with *γ*-aminobutyric acid, and an inverse relationship between *B. thermophilum* and taurochenodeoxycholic acid. Nonetheless, interpretation is limited by the scope of the Quant 500 XL panel, which, while being a quantitative analysis, targets predominantly lipid metabolites, and by the lack of annotation for several pig-specific taxa and compounds not covered in AGORA2, such *as Lactobacillus porci* and *Pseudoscardovia porci.*^95^

Despite the absence of overt metabolic alterations, early correlations emerged between microbial shifts and host traits such as fasting insulin, HOMA-IR, and adiposity. In MFD-fed pigs, reduced *Coprococcus catus* abundance in the duodenojejunum was associated with higher fasting insulin levels and altered indole derivative concentrations in the same segment. Conversely, several *Corynebacterium* species enriched in LFD-fed animals and linked to metabolites such as xanthine and sarcosine were inversely correlated with insulin, body weight, and abdominal circumference, which is consistent with prior findings in mice and with strains from the stools of human patients.^71^^,^^96^ Additional associations reinforce the potential early influence of the small intestinal microbiota on host phenotypes, including the link between *L. johnsonii*, HDL-cholesterol, and hypoxanthine,^68^^,^^97^ and the association of *B. wexlerae*, a proposed antiobesity commensal, with lower HOMA-IR and body weight, alongside changes in bile acids and ceramide species.^70^

These findings support the concept that microbial alterations in the proximal small intestine can influence downstream microbial and metabolic configurations. For example, we observed an increase in known succinate-producing taxa, such as *Fusicatenibacter* in the upper segments, followed by enrichment of succinate-consuming bacteria in distal sites, including *Clostridium* spp. and *D. succinatiphilus*, species implicated in bile acid remodeling, weight gain, and inflammation.^86^^,^^98-102^ These findings were supported by gene analysis of the bacteria in each segment using the Humann pipeline.^32^ Succinate itself has emerged as a microbiota-derived signaling metabolite that can influence host metabolism via SUCNR1 (GPR91)-mediated pathways, promoting gluconeogenesis, adipose tissue remodeling, and immune responses.^102-104^ Thus, the spatial redistribution of succinate dynamics along the gut may reflect a microbially driven metabolic reprogramming cascade initiated by a moderate dietary lipid increase.

The pig represents a highly relevant translational model due to its similarities with humans in terms of gastrointestinal morphology, microbiota composition, and dietary physiology.^7^^,^^105^ Several phenotypic responses observed in pigs closely mirror those described in humans, such as the increase in circulating HDL following a MUFA-rich diet or even the rise in CRP levels, which is particularly pronounced in females with obesity.^91^^,^^106^ Although direct metabolomic comparisons with the human small intestine remain limited by accessibility constraints and methodological differences between analytical platforms, our findings are consistent with some published human data. For example, MFD-fed pigs exhibited increases in nitrotyrosine and decreases in alkaloids and stachydrine, which parallel metabolomic alterations reported in humans.^60^^,^^107^^,^^108^

Regarding the microbiota, while certain species are specific to the porcine gut, our results show strong homologies with studies conducted in the human small intestine, reinforcing the translational value of our model.^95^ In both humans and pigs, microbial load increases markedly from proximal to distal segments, reaching comparable densities (10⁷ CFU/mL in jejunum vs. 10¹¹ CFU/mL in colon), reflecting environmental gradients in pH, bile concentration, oxygen, nutrient availability, and mucus.^7^^,^^15^ Despite species-specific differences in taxonomic distribution, the dominant genera in this intestine region are largely shared, including *Corynebacterium, Actinomyces, Staphylococcus, Veillonella, Klebsiella, Eubacterium,* and *Streptococcus.*^109^ Moreover, alterations in the duodenojejunal microbiota associated with obesity in humans, including our previous observations, also show notable parallels with our pig observations, such as enrichment in *Streptococcus* and *Megasphaera* and depletion in *Corynebacterium, Enterococcus, Blautia,* and *Escherichia.*^19^^,^^83^^,^^110^ However, studies in humans remain scarce and are often limited by the invasive nature of sampling, small cohort sizes, and limited to genus level in metagenomic analyses. Together, these similarities and limitations emphasize the suitability of the pig as a translational model for studying diet-induced alterations of the small intestine and their implications for host phenotype and metabolic health.

While our findings reveal early diet-driven ecosystem shifts, they also highlight the need for mechanistic studies to validate causal relationships and decipher the mechanisms involved. In fact, the cross-sectional nature of the study design does not allow for establishing direct causality behind the multiple associations emerging from multiomics statistical analyses. Despite our *in silico* modeling framework for studying bacterial metabolism via AGORA2 and using data from the literature, this provides some mechanistic support in some of the recovered associations, and other *in vivo* mechanistic studies would be necessary to validate the results. This will allow to strengthen our knowledge of the links between the small intestinal microbiota and the modulation of physiological parameters, which could lead to the development of possible treatments for metabolic diseases. Many of the observed correlations, such as *L. reuteri*'*s* linked with epithelial repair via AhR activation^111^^,^^112^ or *E. coli*'s regulation of tight junctions,^113^ suggest that microbial metabolites are key regulators of host intestinal functions. Future studies should aim to isolate microbial strains and track their metabolic outputs *in vitro* and *in vivo*, especially in the underexplored small intestine.

In summary, this study provides a comprehensive spatial and functional map of diet-induced alterations in the gut ecosystem before metabolic disease onset. Our findings underscore the proximal small intestine as a key site of early microbial and metabolic modulation by the diet, highlight the relevance of the Iberian pig as a preclinical model, and open avenues for future studies targeting microbiota-mediated prevention of metabolic diseases.

## Author contributions

Conception & design: AR, MVG, TLR, KC. Perform the experiments: JD, RA, PT, CR, TLR, MVG, AR, OV, DCP, and AGB. Resources: MVG, TLR, KC. Data curation: AR, MVG, CR, PT, TLR, JD, OV, RA, EB. Data analysis: AR, EB. Technical & material support: AM, FM, KCh. Supervision: GM, TLR, KC, KCh. Writing-original draft: AR. Writing- review & editing: AR, TLR, KC. All authors have read and approved the current version of the manuscript.

## Supplementary Material

Supplementary material Supplemental tables

Supplementary material Figure S1

Supplementary material Figure S2

Supplementary material Figure S3

Supplementary material Figure S4

Supplementary material Table 7a

Supplementary material Table 7b

## Data Availability

The data that support the findings of this study are openly available in European Nucleotide Archive at http://www.ebi.ac.uk/ena/browser/view/PRJEB89232, reference number PRJEB89232 and in Zenodo repository at http://zenodo.org/records/15772056 (DOI 10.5281/zenodo.15772055).
