# Supplementary figures and images for: Moderate increase in dietary fat induces alterations of microbiota and metabolome along the digestive tract prior to systemic metabolic changes: insights from a pig model
Source: Gut Microbes. 2025 Dec 1;17(1):2587964. doi: 10.1080/19490976.2025.2587964 (PMC12674348; doi:10.1080/19490976.2025.2587964)

Males

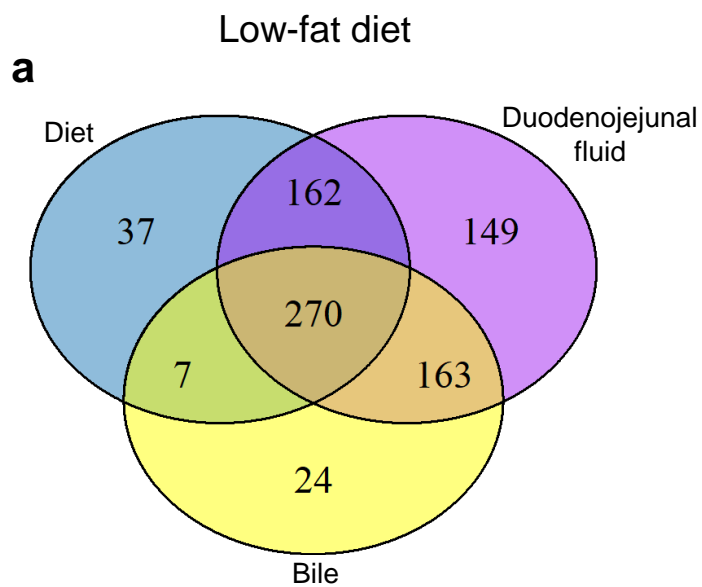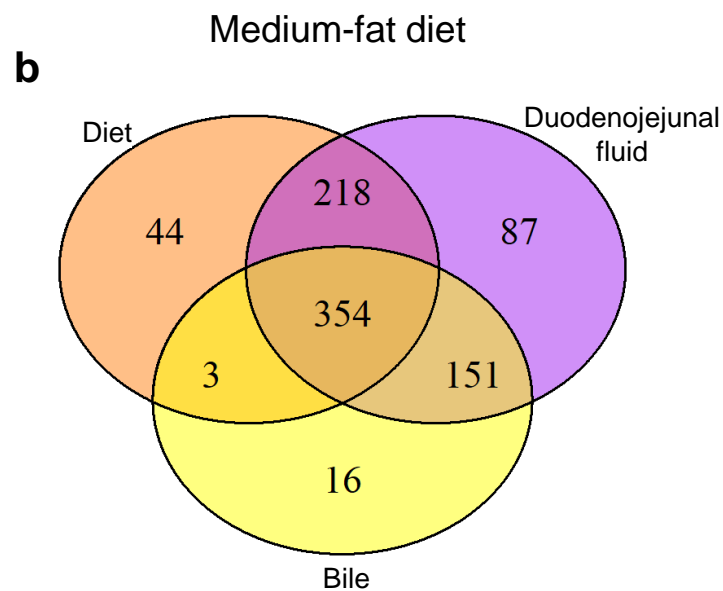

Females

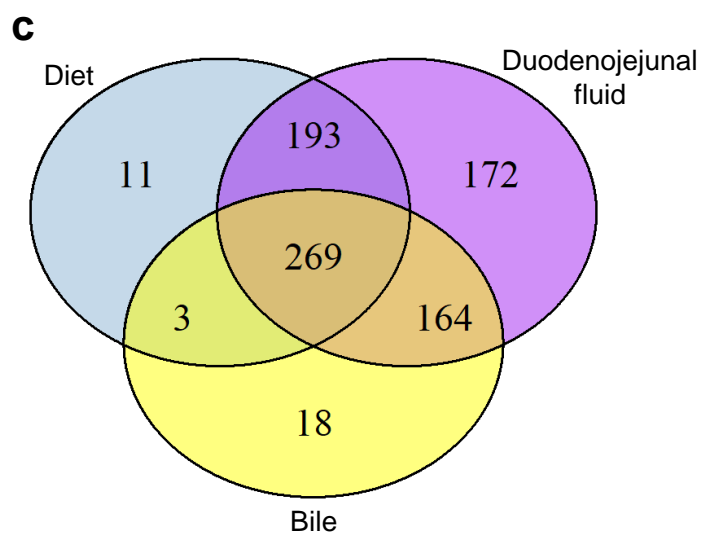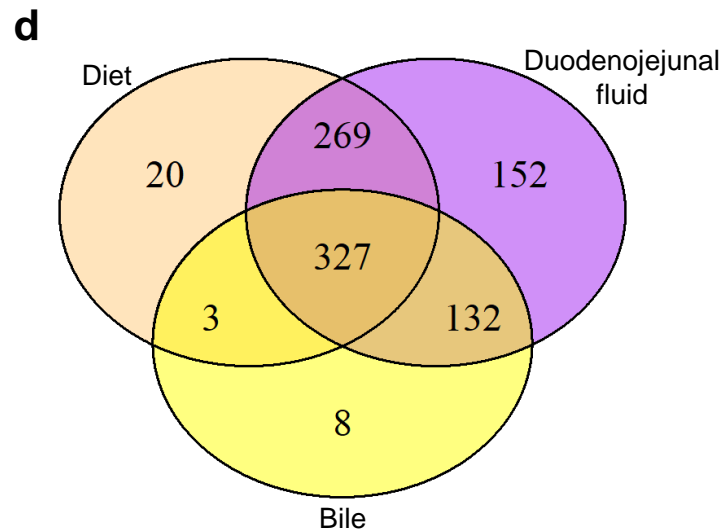

Supplement: Supplementary material — Figure S1 [file KGMI_A_2587964_SM5176.pdf]

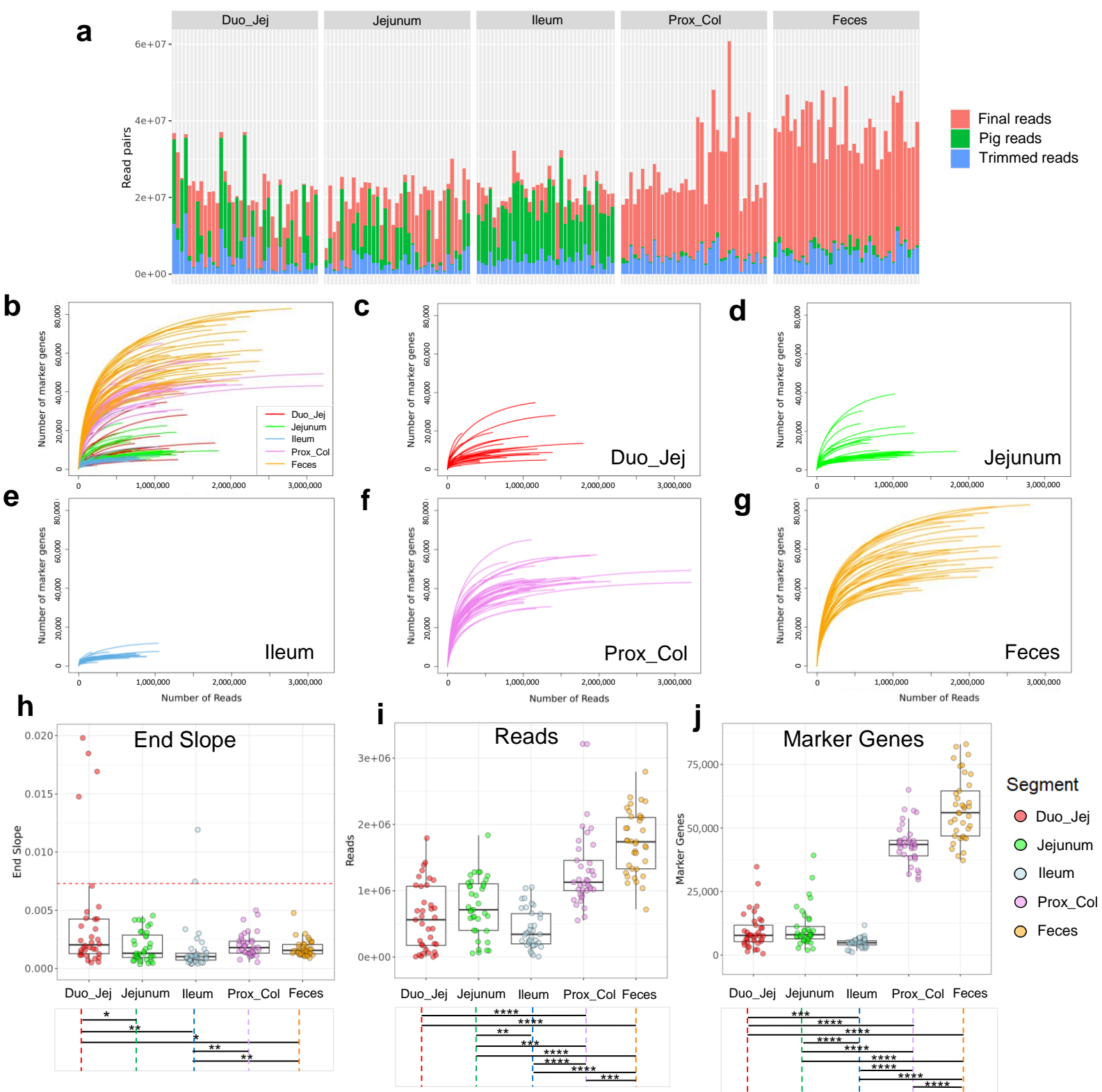

Supplement: Supplementary material — Figure S2 [file KGMI_A_2587964_SM5171.pdf]

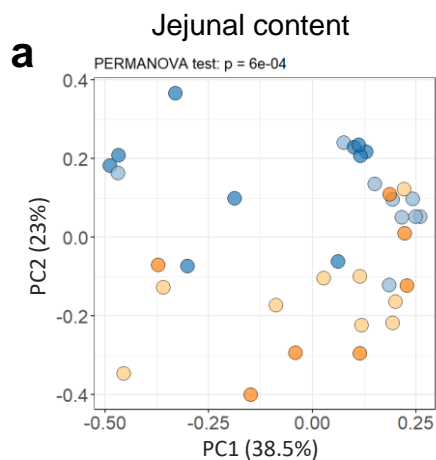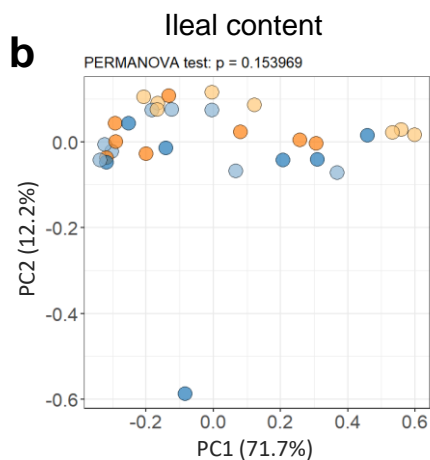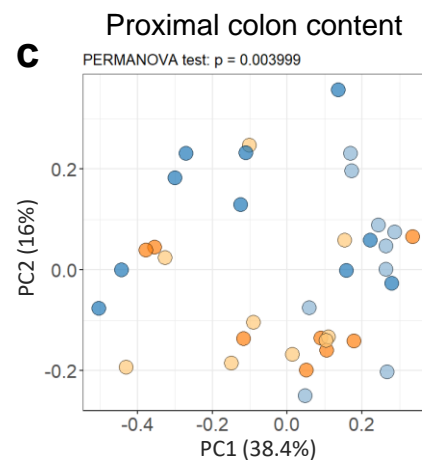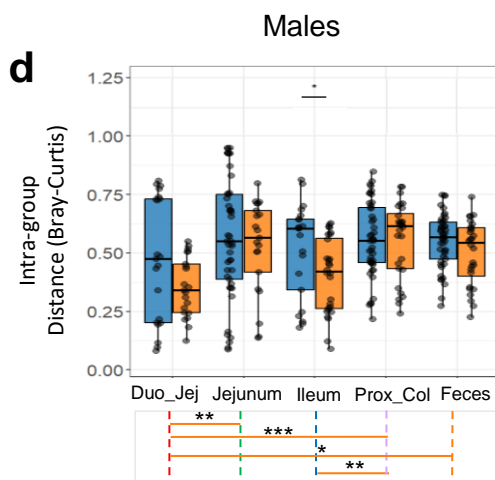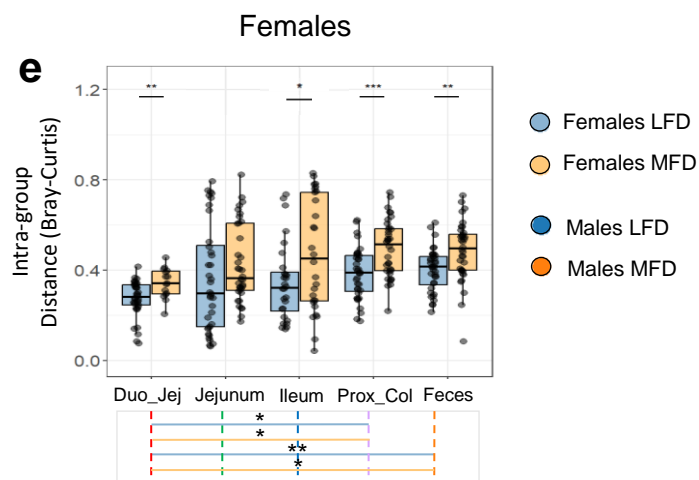

● Females LFD  
● Females MFD  
● Males LFD  
● Males MFD

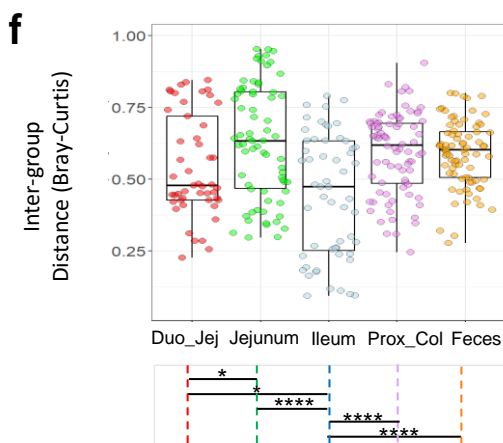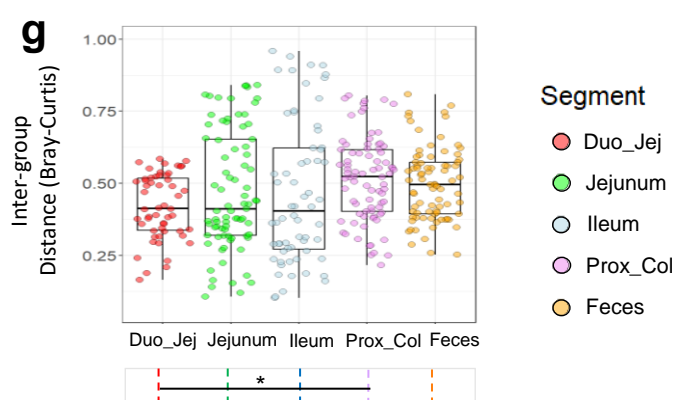

Segment

● Duo\_Jej  
● Jejunum  
● Ileum  
● Prox\_Col  
● Feces

Supplement: Supplementary material — Figure S3 [file KGMI_A_2587964_SM5175.pdf]

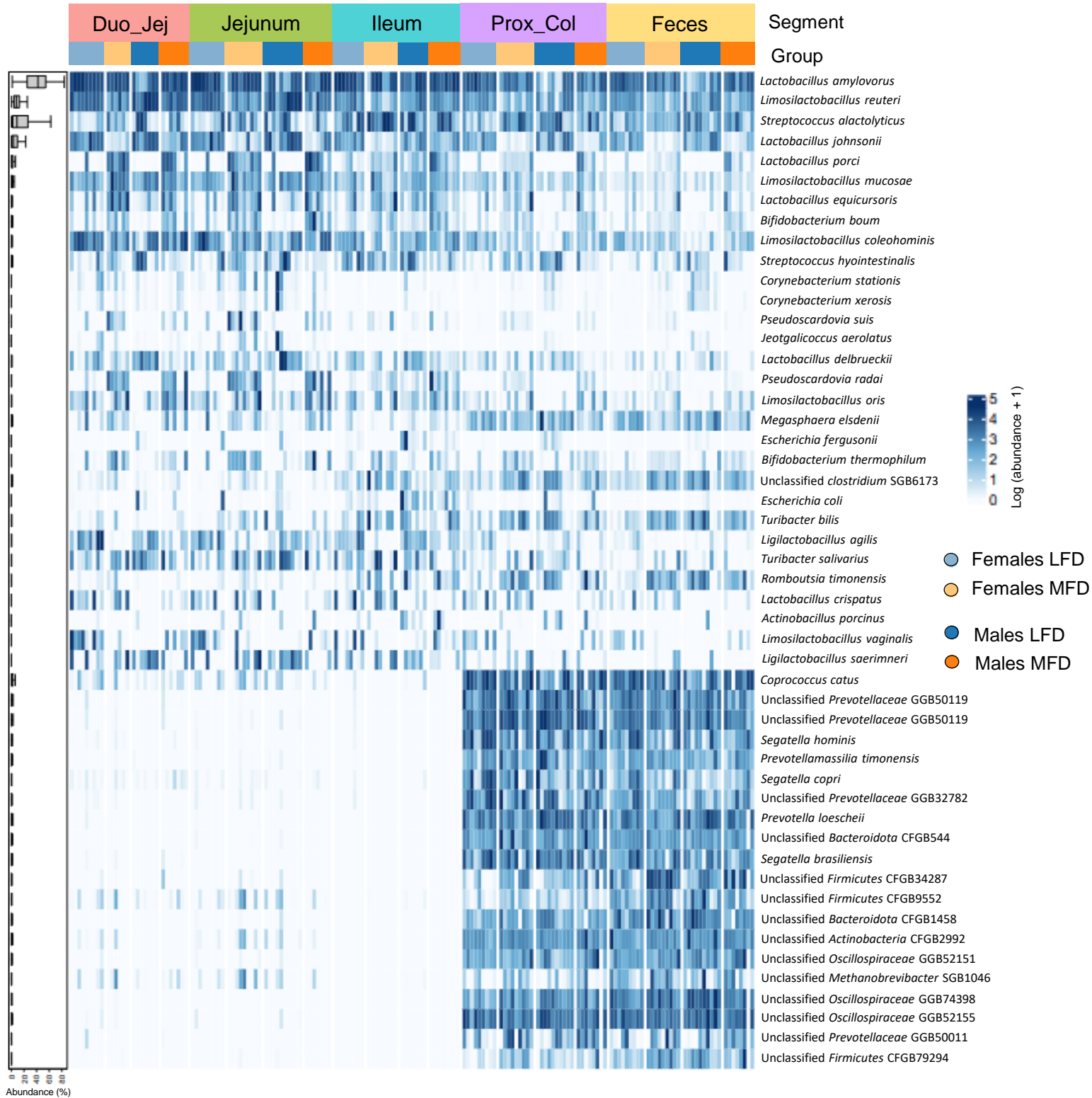

Supplement: Supplementary material — Figure S4 [file KGMI_A_2587964_SM5172.pdf]
